# White-tailed deer are a biotic filter during community assembly, reducing species and phylogenetic diversity

**DOI:** 10.1093/aobpla/plu030

**Published:** 2014-06-09

**Authors:** Danielle R. Begley-Miller, Andrew L. Hipp, Bethany H. Brown, Marlene Hahn, Thomas P. Rooney

**Affiliations:** 1 Department of Ecosystem Science and Management, The Pennsylvania State University, University Park, PA 16802, USA; 2 The Morton Arboretum, Lisle, IL 60532, USA; 3 Department of Biological Sciences, Wright State University, Dayton, OH 45435, USA

**Keywords:** Browsing, herbivory, phylogenetic clustering, phylogenetic community ecology, plant–animal interactions, species diversity.

## Abstract

Community assembly entails a filtering process, where species found in a local community are those that can pass through environmental (abiotic) and biotic filters and successfully compete. Previous research has demonstrated the ability of white-tailed deer (*Odocoileus virginianus*) to reduce species diversity and favour browse-tolerant plant communities. In this study, we expand on our previous work by investigating deer as a possible biotic filter altering local plant community assembly. We used replicated 23-year-old deer exclosures to experimentally assess the effects of deer on species diversity (*H*′), richness (SR), phylogenetic community structure and phylogenetic diversity in paired browsed (control) and unbrowsed (exclosed) plots. Additionally, we developed a deer-browsing susceptibility index (DBSI) to assess the vulnerability of local species to deer. Deer browsing caused a 12 % reduction in *H*′ and 17 % reduction in SR, consistent with previous studies. Furthermore, browsing reduced phylogenetic diversity by 63 %, causing significant phylogenetic clustering. Overall, graminoids were the least vulnerable to deer browsing based on DBSI calculations. These findings demonstrate that deer are a significant driver of plant community assembly due to their role as a selective browser, or more generally, as a biotic filter. This study highlights the importance of knowledge about the plant tree of life in assessing the effects of biotic filters on plant communities. Application of such knowledge has considerable potential to advance our understanding of plant community assembly.

## Abstract (precis)

Promotional Statement: White-tailed deer browsing has been implicated in the loss of species diversity from forests throughout eastern North America. We build on this previous research by examining how browsing also affects phylogenetic community structure. With this approach, we can better understand the role of deer browsing in the community assembly process. In browsed plots, we found that reductions in phylogenetic diversity were much greater than reductions in species richness or diversity. Species persisting in browsed communities were also closely-related. Our findings indicate deer browsing acts as a biotic filter during the community assembly process.

## Introduction

During the community assembly process—the formation of local communities from a regional species pool—most available species are ‘filtered out’ of local communities on the basis of genotypes, dispersal limitations or sets of traits that are least suited to a particular habitat (Keddy 1992; HilleRisLambers *et al.* 2012). The species found in a local community are those that can pass through environmental (abiotic) and biotic filters and successfully compete. Herbivores may act as a biotic filter (Augustine and McNaughton 1998; Suzuki *et al.* 2013) by preventing a species that is otherwise well adapted to the abiotic conditions in a local community from persisting over time. Herbivory is thus expected to produce local communities consisting of species with traits that confer resistance to or tolerance of herbivory. If herbivory resistance or tolerance evolves on the plant tree of life (i.e. if it is phylogenetically heritable), we would expect herbivory to alter phylogenetic diversity within communities. Such changes are important from both a theoretical and applied perspective: understanding shifts in the phylogenetic structure of plant communities in response to experimental removal from herbivory can help elucidate how and to what extent herbivory is a biotic filter shaping plant communities. Phylogenetic diversity needs to be better investigated as a tool for more targeted conservation efforts and for understanding the maintenance of biodiversity in conservation areas (Faith 1992).

White-tailed deer (*Odocoileus virginianus*) overabundance is a conservation issue throughout parts of eastern North America, because browsing alters community structure, composition and diversity of forests (Horsley *et al.* 2003; [Bibr PLU030C10][Bibr PLU030C10]). Changes in community composition reflect the selective browsing strategy of white-tailed deer. Deer consume palatable, nutrient-rich species when available and lesser quality browse when high-quality sources are depleted (Beals *et al.* 1960; Balgooyen and Waller 1995; Waller and Alverson 1997; [Bibr PLU030C10][Bibr PLU030C10]). Unpalatable, browse-tolerant and non-preferred plant species are commonly observed in heavily browsed areas (Tremblay *et al.* 2006; Rooney 2009; Martin *et al.* 2010; Royo *et al.* 2010; Goetsch *et al.* 2011). It is thus not surprising that deer browsing has been linked to both plant population extirpations and reductions in forest understorey species richness (SR) (Rooney and Dress 1997; Horsley *et al.* 2003; Rooney *et al.* 2004; Martin *et al.* 2010).

Here, we examine a community in which deer have been experimentally removed for two decades and investigate how this has affected the phylogenetic community structure. In this study, we surveyed vascular plant taxa in successive years in paired control and deer exclosure plots. We had three main goals of our analysis. We first determined the effects of deer browsing on community structure by comparing both species and phylogenetic diversity in control and exclosed areas. We were particularly interested in whether phylogenetic data provided additional information not contained in species diversity measures (Vellend *et al.* 2011), and whether deer browsing altered the degree of phylogenetic relatedness within each local community. We next tested for phylogenetic patterns in two categories of traits associated with vulnerability to deer browsing: browse type (Rooney 2009) and pollination mode (Rooney *et al.* 2004). We then developed a deer-browsing susceptibility index (DBSI) to quantitatively separate vulnerable from non-vulnerable species at our study site. By applying the tools of phylogenetic diversity to an applied study of the conservation impacts of deer herbivory, we refine our understanding of how deer serve as a biotic filter in plant community assembly.

## Methods

### Study site

This study was conducted at the 2500 ha Dairymen's Club in Wisconsin, USA (46.15°N, 89.68°W). The site is privately owned and managed for conservation, recreation and scientific research. The climate is continental, with average yearly precipitation between 550 and 780 mm and a mean temperature range of −20 °C in winter to 32 °C in summer (Rooney *et al.* 2004). The landscape is heterogeneous, including lakes, sedge meadows and mixed conifer–hardwood forest. Dairymen's Club purchased the property in 1925, and all hunting has been prohibited since then (Rooney 2006). In the absence of hunting, the deer population grew quickly. Growth was further fuelled by a supplemental deer-feeding programme from 1950 to 2000. In feeding areas, local concentrations exceeded 100 deer km^−2^. Forests are the predominant land cover type in the area, and dominant canopy trees include *Acer saccharum, Tsuga canadensis* and *Betula alleghaniensis*.

### Deer exclosure experiment

In 1990, four deer exclosures were constructed within 500 m of feeding areas on the property to protect vulnerable plant species from continuous browsing. These long-term exclosures are 1.8 m tall, range in size from 196 to 720 m^2^ (Rooney 2009) and are constructed of 2.5 × 7.5 cm wire mesh. The deer densities at this site throughout the 20th century were much higher than were found throughout the northern Wisconsin region (Rooney 2006). To understand the long-term effects of these prolonged deer densities on forest understorey plant communities, three permanent ground-vegetation transects were established inside and outside each exclosure in 2006 (Rooney 2009). Each transect totals 10 m in length and extends 5 m into an adjacent unfenced area (control) and 5 m into its paired exclosure (separated by the exclosure fence). The unique history of deer population dynamics at this study site, combined with the construction of these exclosures decades ago, allows us to better assess the long-term effects of deer as a driver of plant community assembly.

### Vegetation data collection

Per cent cover data were collected from the permanent transects during the first or second week of June each year from 2006 to 2012, except 2007. The line-intercept method was used to obtain cover data. All plants ≤1 m tall were identified to species. Each exclosure and control area was sampled equally, regardless of size of exclosure. Along each transect, a measuring tape was laid on the ground beneath the vegetation. If any part of the organ of a plant (i.e. leaf, stem, flower) intercepted the transect the plant was identified to species, and length (to the nearest cm) of the tape covered was recorded. Per cent cover for the *i*th species in a plot was calculated as (Σ*n*_i_)/1500, where *n* is the length of the tape covered by each occurrence of species *i* (to the nearest cm) along that transect. The denominator is the length in centimetres of three 5 m transects. Because multiple species can intercept the same transect segment at different heights, the total per cent cover can exceed 100 %. Per cent cover of the *i*th species across all four plots within a treatment is (Σ*n_i_*)/6000 (Rooney 2009).

### Diversity metrics and analysis

#### Phylogeny

A rooted phylogenetic tree was created using DNA sequences of three gene regions. The tree is site specific, in that we only obtained gene sequences from species found at the study site. We did not sequence species from the regional species pool not found in our study plots. Of these DNA regions, one (the 5′ end of the chloroplast *rbc*L gene) is highly conserved across angiosperms. It is a widely used DNA barcoding gene (Kress and Erickson 2007) that has been the workhorse of broad-scale phylogenetics across higher plants (e.g. Chase *et al.* 1993). This gene aligns unambiguously across green plants and provides solid information on genetic relationships across the samples we studied. The other two DNA regions are more rapidly evolving and used widely in fine-scale phylogenetics in flowering plants: the chloroplast intergenic spacer between the 3′ end of the *trn*L exon and the 5′ end of the *trn*F exon (hereafter in the paper referred to as the *trn*L–*trn*F region) (Taberlet *et al.* 1991), and nuclear ribosomal internal transcribed spacer regions (ITS1 and ITS2), including the embedded 5.8S gene (hereafter in the paper referred to collectively as the ITS region) (Baldwin *et al.* 1995). These genes, however, were not fine-scale enough to determine intraspecific differences between individuals. All sequences were used to determine genetic differences at the species level, and to confirm species identification in cases of uncertainty. Gene sequences for 18 of the 36 species at our study site were obtained from NCBI GenBank (Benson *et al.* 2010) **[see Supporting Information, bolded]**, and the other 18 were sequenced from material collected during the 2012 field season **[see Supporting Information, italicized]**. Sequences for this study used the following PCR primers: ITS-I (Urbatsch *et al.* 2000) and ITS-4 (White *et al.* 1990); *rbc*La-F (Levin *et al.* 2003) and *rbc*La-R (Kress *et al.* 2009) and for the *trn*L–*trn*F intergenic spacer, Taberlet *et al.* (1991) primers e and f. PCR reactions were conducted as in Hipp *et al.* (2006), with the following cycling regimens: ITS: 94.0° for 5:00; 35 cycles of: 94.0° for 0:30, 48.0° for 1:00, 72.0° for 1:30; 72.0° for 7:00. *rbc*L: 94.0° for 5:00; 35 cycles of: 94.0° for 0:30, 52.0° for 1:00, 72.0° for 1:30; 72.0° for 7:00. *trn*L–*trn*F: 95.0° for 3:00; 50 cycles of: 95.0° for 0:20, 45.0° for 0:30, 52.0° for 4:00; 72.0° for 7:00. PCR products were cycle sequenced using BigDye reaction kits and the PCR primers, and unincorporated dye terminators were removed using CleanSEQ magnetic beads (Agencourt, Beckman Coulter). PCR was conducted at The Morton Arboretum, and sequencing was conducted on an ABI 3730 capillary sequencer in The Pritzker Lab of the Field Museum. Double-stranded DNA sequence contigs were cleaned manually in Sequencher 3.0 (Gene Codes Corporation, Ann Arbor, MI, USA) and exported as text for analysis.

DNA sequences were aligned using Muscle v. 3.8.31 (Edgar 2004*a*, *b*) and manually adjusted. Data for the *rbc*L region were globally aligned without ambiguities, including all taxa. Global alignment of all taxa simultaneously for the ITS and *trn*L–*trn*F regions, however, produced alignments that were riddled with ambiguities. To address this, data matrices were first aligned by APGIII order. Then, profile-to-profile alignments were utilized, in which the alignment within each order is held fixed but nucleotide positions are allowed to shift among orders. Profile-to-profile alignments were conducted among most closely related orders, moving progressively up the tips to the root of the green plants tree of life, using the Angiosperm Phylogeny Group tree (APG III 2009) as updated in APG Web (Stevens 2001 onwards).

**Figure 1.**
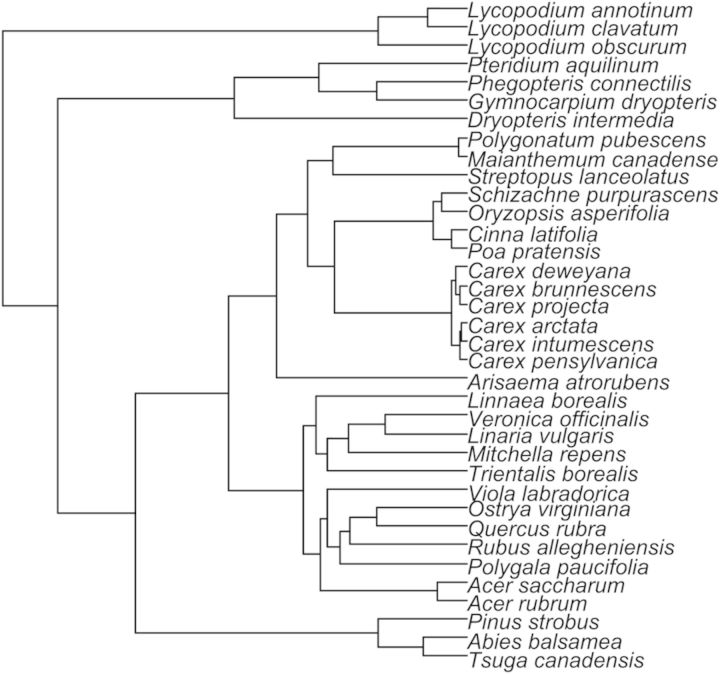
Rooted phylogenetic tree (using ITS, *rbc*L and *trn*L–*trn*F regions) of all species present at the study site. Phylogeny was estimated using maximum likelihood in RAxML, as described in the Methods.

Multiple alignments were then concatenated and analysed under likelihood in RAxML v.7.2.6 (Stamatakis 2006), using the GTRCAT nucleotide substitution model, using the multithreading option on a 4-core Intel processor (Stamatakis and Ott 2008). Analysis was conducted using 200 bootstrap replicates. Branch lengths were optimized on the resulting tree using penalized likelihood (Sanderson 2002) as implemented in the ape package (Paradis *et al.* 2004) of R v.2.13.1 (R Development Core Team 2011). Smoothing parameters from 10 to 0.001 were tried and found to have no appreciable effect on the branch lengths on the tree. The reported tree (Fig. 1) utilizes a smoothing parameter of 1.0. All DNA sequences generated for this study are deposited in NCBI GenBank **[see Supporting Information]**.

#### Phylogenetic diversity

Using the site-specific phylogenetic tree, we analysed phylogenetic signal, diversity and patterns of community structure. To test for phylogenetic signal we used two different approaches with two different character traits: browse type (woody, broadleaf forb, fern, grass, sedge or lycopod) and pollination mode (biotic or abiotic). Based on previous research, we predicted species persisting in browsed areas would be abiotically pollinated grasses and sedges (graminoids) (Rooney *et al.* 2004; Rooney 2009). We estimated the phylogenetic signal of pollination mode (Rooney *et al.* 2004), a binary trait, using Fritz and Purvis’ (2010) *D* statistic in the caper package in R (Orme 2013). A value of *D* = 0 indicates a trait consistent with a Brownian threshold model, while a value of *D* = 1 indicates a trait following a random distribution. Values can fall outside of this range, with those significantly less than 0 indicating high phylogenetic conservatism, and values significantly higher than 1 indicating phylogenetic overdispersion. Significance is assessed by comparing observed trait distributions with expected distributions simulated under a Brownian motion model or by random permutation of the original tip states. For the multistate trait browse type (Rooney 2009), we used Mesquite (Maddison and Maddison 2011) to calculate the minimum number of character transitions needed to observe trait distribution under maximum parsimony on our site-specific maximum likelihood tree (Fig. 1), and compared the observed value with a null distribution calculated over 1000 permutations of the tip states. The Type I error rate (*P*) value was estimated as the number of permutations for which the parsimony score (minimum number of character steps) was less than or equal to the parsimony score for the browse type data.

As a general metric of phylogenetic diversity we used mean pairwise phylogenetic distance (MPD) as implemented in the R package picante (Kembel *et al.* 2013). We chose MPD because it is less correlated with SR than Faith's (1992) phylogenetic diversity (Yessoufou *et al.* 2013) and is more sensitive to changes between distantly related taxa than mean nearest taxon distance. To test for phylogenetic patterns of community structure we used the net relatedness index (NRI, also in picante), which compares the phylogenetic structure of our measured communities with randomly permuted trees. Specifically, we tested if control or exclosure communities were phylogenetically autocorrelated (clustered), showing less phylogenetic diversity than expected at random. Large positive NRI values indicate clustering, while a large absolute value derived from a negative NRI indicates phylogenetic overdispersion.

#### Species diversity

Species richness was defined as the total number of species encountered along transects at each plot. Species diversity indices were calculated using the Shannon–Wiener (Shannon and Weaver 1949) standard diversity metric (*H*′). We used *H*′ because it is weighted for abundance and is less correlated with SR than Simpson's diversity index (*D*). Per cent cover data for each species from each control and exclosure plot was used for abundance.

### Statistical analysis

We compared MPD, SR and *H*′ of controls and exclosures using a linear mixed effects model with site as a random effect, and treatment, year and treatment/year interaction as fixed effects. The random effect of site was retained within the model if *P* < 0.10. Significant fixed effects were reported at the *P* < 0.05 level. For NRI, we pooled all sites together by treatment and analysed by year, comparing phylogenetic distance of communities across the most parsimonious tree with simulated ones with shuffled tip labels over 999 permutations. This shuffling simulates a null expectation of no effect of phylogeny. Resulting *P* values were used to identify which years and which exclosures were most influenced by phylogenetic structure.

### Deer-browsing susceptibility index

To evaluate plant species susceptibility to deer browsing and identify those reliant on exclosures for persistence, we developed a DBSI. It compares relative cover of plant species inside and outside exclosures, and scales from 0 to 1 for each species. The DBSI value for each species represents the fraction of that species' per cent cover inside versus outside the exclosure. A score of 0 indicates a species is only found outside exclosures, while a score of 1 indicates a species is only found inside exclosures. To exclude rare species that would skew DBSI calculations, we only included species present in more than two exclosures or controls in two or more years. Deer-browsing susceptibility index was calculated separately for each species in each year using the following equation:

$$\text{DBSI}=\left(\sum C_{e}\right)/\left(\sum C_{e}+\sum C_{c}\right)$$

where *C*_e_ is the per cent cover inside the exclosure, *C*_c_ is the per cent cover outside the exclosure and Σ(*s* = 1,2,…,*n*) is the sum of *C*_e_ (or *C*_c_) across all exclosures (or controls). In the results, we report a single mean DBSI value across all years for each species.

## Results

### Species and phylogenetic diversity

**Figure 2.**
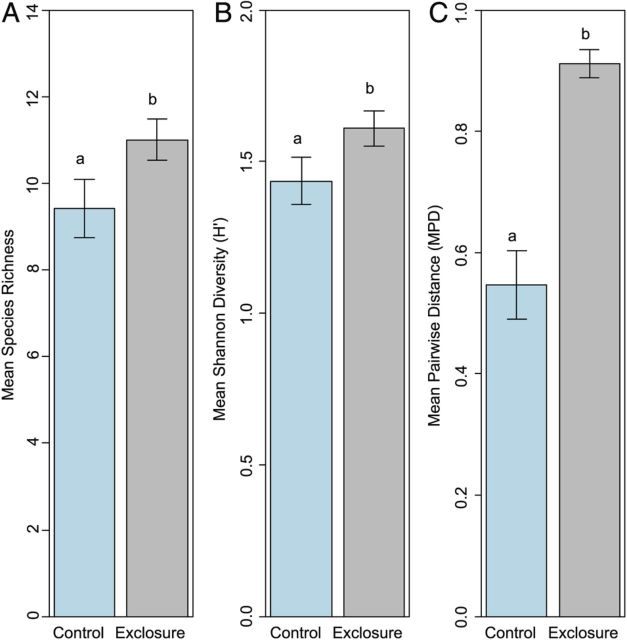
Mean species richness (A), Shannon–Weiner diversity (B) and mean pairwise phylogenetic distance (C) in exclosure (grey bars) and control (blue bars) areas across all years (2006–2012, excluding 2007). Different letters indicate statistical significance between groups at the *P* < 0.05 level, as tested using ANOVA in a linear mixed effects model. Error bars are ±1 SE.

Species richness and *H*′ responded similarly to browsing from deer. Browsing caused a 17 % reduction in SR (*F*_(1,43)_ = 5.81, *P* = 0.02; Fig. 2A) and 12 % reduction in *H*′ (*F*_(1,42.99)_ = 4.43, *P* = 0.04; Fig. 2B). Neither metric showed a year (SR: *F*_(5,38)_ = 0.189, *P* = 0.96; *H*′: *F*_(5,37.99)_ = 0.658, *P* = 0.66), or treatment/year interaction effect (SR: *F*_(5,33)_ = 0.913, *P* = 0.48; *H*′: *F*_(5,32.99)_ = 0.468, *P* = 0.7971). Site was significant in both models (SR: *χ*^2^_(1,48)_ = 11.48, *P* = 0.0007; *H*′: *χ*^2^_(1,48)_ = 7.52, *P* = 0.0061).

**Figure 3.**
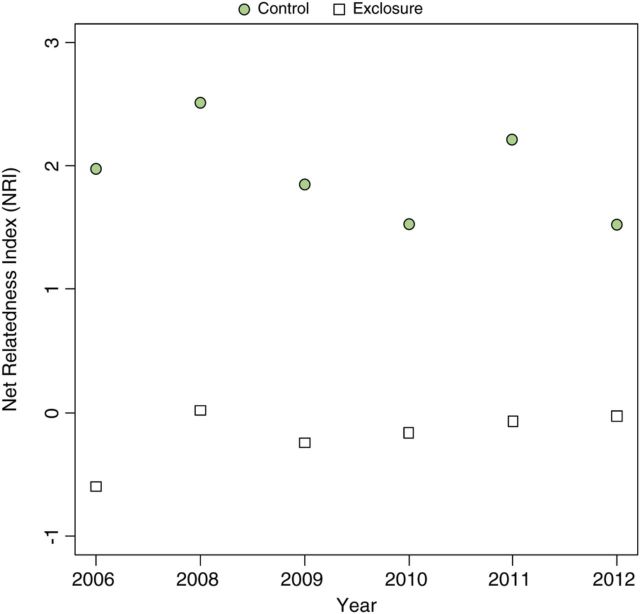
Net relatedness index of exclosure areas (squares) and control areas (circles) from 2006 to 2012 (excluding 2007). Points highlighted in green indicate NRI values significantly greater than expected by chance (*P* < 0.05, based on 999 random permutations of the tip states on the phylogeny).

Browsing significantly reduced phylogenetic diversity (MPD) by 63 % (*F*_(1,42.98)_ = 42.36, *P* < 0.00001; Fig. 2C). The effect of year (*F*_(5,37.98)_ = 0.24, *P* = 0.94) and the interaction of year and treatment (*F*_(5,32.98)_ = 0.46, *P* = 0.80), however, were not significant in browsed or unbrowsed plots. Site was a significant random effect in the model at the *P* < 0.10 level (*χ*^2^_(1,48)_ = 2.72, *P* = 0.099). Analysis of NRI showed significantly higher relatedness in browsed areas than expected by chance in each year sampled (range: +1.53 to +2.51, *P* < 0.05; Fig. 3). Exclosure areas did not show structured phylogenetic response (range: −0.60 to +0.01, *P* > 0.05).

### Phylogenetic signal

Pollination mode exhibits clustering (*D* = −0.672; Table 1) relative to a random null model (two-tailed *P* < 0.001; 1000 permutations of the tip states). Clustering is also stronger than expected under Brownian motion null distribution, but not significantly so (two-tailed *P* = 0.14; 1000 Brownian motion simulations of the tip states under a threshold model; Fritz and Purvis 2010). Browse type (woody, broadleaf herb, fern, grass, sedge or lycopod) also showed significant phylogenetic clustering relative to a phylogenetically neutral null model (Table 1). The number of evolutionary steps in the maximum parsimony reconstruction of browse type was 8, while the mean number of evolutionary steps in 1000 simulated trees with permutated tips was 22.1 (95 % CI [19, 25], *P* < 0.001).

**Table 1.** Tests for phylogenetic signal for pollination mode and browse type using Fritz and Purvis' *D* statistic and phylogenetic autocorrelation, respectively. To test for phylogenetic signal for the binary trait pollination mode, we used Fritz and Purvis' *D* statistic. For this test, significance is assessed by comparing observed trait distributions with expected distributions simulated under a Brownian motion model or by random permutation of the original tip states. For the multistate trait browse type we calculated the maximum parsimony of browse type on our site-specific maximum likelihood tree, and compared the observed value with a null distribution generated by 1000 permutations of the tip states. The Type I error rate (*P*) value for this test was estimated as the minimum number of simulated trees with parsimony reconstruction of less than or equal to the number of steps in the observed reconstruction. Significant *P* values (*P* < 0.05) for both tests indicate that traits are phylogenetically clustered (i.e. not phylogenetically independent).

Parameter	Phylogenetic autocorrelation	Parameter	Fritz and Purvis' *D* statistic
Character type	Categorical (browse type)	Character type	Binary (pollination mode)
Number of permutations	1000	Number of permutations	1000
Difference in no. of evolutionary steps (MP−mean shuffled)	−14.1	*D* statistic	−0.674
95 % confidence interval	LCI: 19UCI: 25	Probability of *D* given Brownian phylogenetic structure	0.144
*P* value	<0.001	Probability of *D* given random phylogenetic structure	<0.001

### Deer-browsing susceptibility index

**Figure 4.**
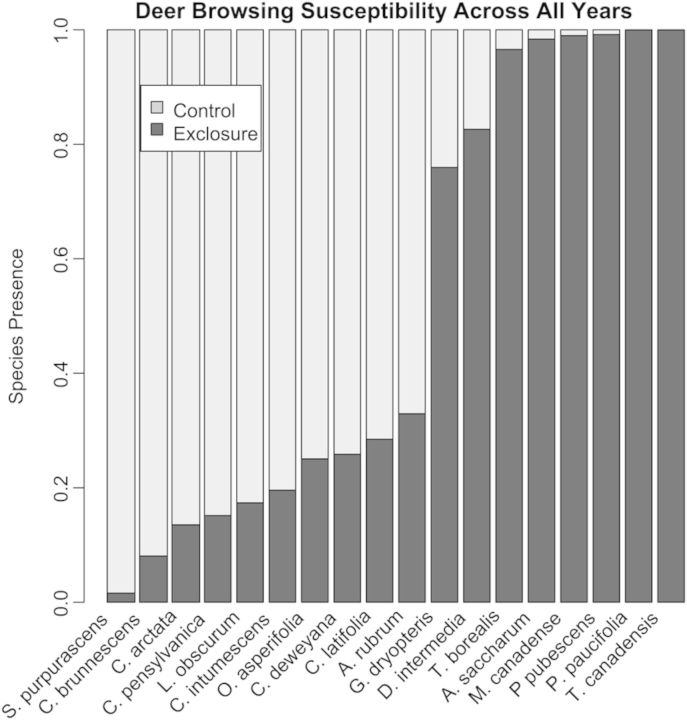
A stacked bar graph representing the mean calculated DBSI of each species present in more than one exclosure in two or more years. Light grey bars indicate the proportion of the species present in control areas, while dark grey bars indicate the proportion of the species present in exclosures. Species are ordered left to right from the least susceptible (*S. purpurascens*) to most susceptible (*T. canadensis*).

The two most deer-browse-susceptible species (*Polygala paucifolia* and *T. canadensis*) were found exclusively inside exclosures (DBSI = 1.0; Fig. 4). Both species represent very different growth patterns, as *P. paucifolia* is a perennial broadleaf herb and *T. canadensis* is an evergreen woody-browse species. All species in the low susceptibility category were graminoids or club mosses, with *Schizachne purpurascens* being the least susceptible of all species analysed (DBSI = 0.02; Fig. 4).

## Discussion

### Species and phylogenetic diversity

Excluding deer for two decades significantly increased species diversity, richness and phylogenetic diversity of plant communities. These results are consistent with previous studies documenting the loss of species diversity of plant communities due to selective browsing by deer (Gill and Beardall 2001; Horsley *et al.* 2003; Rooney *et al.* 2004; Suzuki *et al.* 2013). These results also reveal a substantial loss of phylogenetic diversity in browsed plots. If we set the basal node of our ultrametric tree to a depth of 432 million years, based on a recent estimate of the age of the Tracheophyta (Smith *et al.* 2010), the observed loss of only a few branches represents an average difference in phylogenetic diversity between browsed and unbrowsed plots of 372.5 million years.

We found significant changes to the phylogenetic community structure of browsed plant communities. These findings demonstrate the ability of deer to shape northern plant communities by filtering out species that share a suite of phylogenetically heritable traits contributing to their browse susceptibility. This conclusion rests on two findings. First, pollination mode and browse type (two traits linked to plant-browsing susceptibility) exhibit significant phylogenetic signal. Deer are thought to visually cue in on biotically pollinated plants due to their conspicuous flowers (Wiegmann and Waller 2006), and since ungulate browse type classes are taxonomically clustered, we expected this trait to exhibit a strong phylogenetic signal. As we observe in this study, many other studies report increases in the relative abundances of the grass and sedge browse types following increases in deer density (Halls and Crawford 1960; Kie *et al.* 1980; Horsley *et al.* 2003; Rooney *et al.* 2004). We did not, however, find an increase in the relative abundance of the fern browse type in response to deer browsing, contrasting findings elsewhere in North America and New Zealand (Royo and Carson 2006).

Second, plant community phylogenetic patterns showed significant phylogenetic clustering in browsed plots across all years, indicating species in these communities were much more related than expected by chance. Unbrowsed plots showed no phylogenetic pattern relative to the suite of species observed. We interpret phylogenetic clustering in browsed communities as arising from a biotic filtering process during community assembly. Because each pair of browsed and unbrowsed plots is spatially adjacent in homogeneous environments, it is unlikely that abiotic environmental filtering accounts for phylogenetic clustering in browsed plots (Mayfield and Levine 2010). It is more likely that any species from our species pool can establish in browsed plots, but that many are competitively excluded due to fitness inequalities arising from their low browse tolerance or resistance (Chesson 2000; Mayfield and Levine 2010). Deer browsing favours species from the flowering plant clade Poales, represented in our study by the Poaceae and Cyperaceae, thus filtering out much phylogenetic diversity. This effect is reflected both in the phylogenetic patterns of trait conservatism and in our NRI values. The consequence is the loss of representative evolutionary history in the forest understorey layer.

### Deer-browsing susceptibility index

In this study, species in the low deer-browsing susceptibility category exhibited characteristics of tolerant or resistant species. Eight species were of either the grass or sedge (graminoid) browse type with abiotic pollination mode. Collectively they are considered browse tolerant, because they have basal meristems and are able to regrow following browsing (Coughenour 1985). As expected, species categorized as broadleaf herbs and woody browse types were classified as susceptible, and most of these species exhibit biotic pollination. The more-susceptible broadleaf herbaceous plant species are broadly distributed through the phylogeny, consistent with our NRI values.

Generally, our findings are consistent with other studies showing the ability of deer to promote browse tolerant and unpalatable species (Horsley *et al.* 2003; [Bibr PLU030C10][Bibr PLU030C10]; Royo and Carson 2006). In this study, however, DBSI is based on species presence inside and outside exclosures. It does not directly measure a species' susceptibility to deer browsing based on chemical composition or deer preference. As a result, DBSI could reflect a species' response to certain environmental conditions (e.g. shading and competition). To the extent that deer exclusion creates a more favourable microhabitat for species, DBSI can reflect differences not directly due to deer herbivory.

## Conclusions

In our study area, deer herbivory acts as a biotic filter. Deer reduce species diversity, SR and phylogenetic diversity by filtering out species that have browse-susceptible traits. Deer have suppressed browse-intolerant species and promoted the coexistence of closely related browse-tolerant species. Phylogenetic diversity indices have been previously used as a way to assess effects of disturbance (Cavender-Bares and Reich 2012) or environmental gradients (Pellissier *et al.* 2012) on community structure. Our study is the first to identify white-tailed deer as a significant driver of plant community assembly using phylogenetic methods.

We gain two fundamental insights from applying the tools of phylogenetic community ecology to this classic study system. First, evolutionary history shapes plant responses to herbivory. The traits we measured—and, presumably a host of other unmeasured traits that affect browse-susceptibility—have high phylogenetic heritability. Second, the phylogenetic heritability of these traits shapes the effect of browsing on phylogenetic diversity and community structure. Thus, as has been shown in other studies of herbivory (e.g. Pearse and Hipp 2009, 2012), phylogenetic heritage integrates over a large number of traits and may thus be a better predictor of herbivore susceptibility than even suites of measured traits. There are hundreds of published studies that have used exclosures to examine the influence of deer herbivory on plant community composition. A re-analysis of data from these studies using the framework of phylogenetic community ecology may provide us even stronger evidence about the utility of phylogeny for predicting plant community responses to management and disturbance. It is our expectation that the resulting increased ability to identify species at risk will enable more effective conservation management and further advances in our understanding of plant community assembly.

## Sources of Funding

Our work was funded by Dairymen's Inc. (USA) and a Research Incentive Fund from the Wright State University.

## Contributions by the Authors

D.R.B.-M. collected data, analysed data, drafted first version of the manuscript. A.L.H. conducted analysis and collaborated in interpretation of results and manuscript preparation. B.H.B. and M.H. conducted DNA sequencing and phylogenetic analysis and helped with manuscript preparation. T.P.R. secured funding, collected data, helped with data analysis and contributed to writing the manuscript.

## Conflicts of Interest Statement

None declared.

## Accession Numbers

All novel sequences for nucleic acids have been submitted and accepted into GenBank as of 18 December 2013. A table of accession numbers for these sequences is included in the supporting information section of this manuscript.

## Supporting Information

The following **Supporting Information** is available in the online version of this article –

**File 1.** Accession numbers as approved by GenBank on 18 December 2013. Herbarium voucher numbers pending. NA indicates the gene was not sequenced, bold numbers indicate the gene was obtained from GenBank and numbers in italics indicate the gene was sequenced by The Morton Arboretum.

Additional Information
