# Supplementary material for: White-tailed deer are a biotic filter during community assembly, reducing species and phylogenetic diversity
Source: AoB Plants. 2014 Jun 9;6:plu030. doi: 10.1093/aobpla/plu030 (PMC4078168; doi:10.1093/aobpla/plu030)
Supplement: Additional Information [file supp_6_plu030_index.html]

White-tailed deer are a biotic filter during community assembly, reducing species and phylogenetic diversity — White-tailed deer are a biotic filter during community assembly, reducing species and phylogenetic diversity — Additional Information 

# White-tailed deer are a biotic filter during community assembly, reducing species and phylogenetic diversity

## Additional Information

Additional Information

**Files in this Data Supplement:**

- Additional Information - doc file
