# Supplementary material for: White-tailed deer are a biotic filter during community assembly, reducing species and phylogenetic diversity
Source: AoB Plants. 2014 Jun 9;6:plu030. doi: 10.1093/aobpla/plu030 (PMC4078168; doi:10.1093/aobpla/plu030)
Supplement: Additional Information [file supp_plu030_plu030supp.docx]

| ***Species*** | ***ITS*** | ***rbcL*** | ***trnL-F*** |
| --- | --- | --- | --- |
| *Abies balsamea* | **EF057709** | **JN935605** | **JN935657** |
| *Acer rubrum* | **AF241502** | **HQ589932,** *KF977466* | *KF977443* |
| *Acer saccharum* | **EU720502** | **DQ978432** | **AF401173** |
| *Arisaema atrorubens* | *KF977433* | *KF977468* | *KF977445* |
| *Carex arctata* | **DQ998900** | **HQ589986** | **DQ998953** |
| *Carex brunnescens* | **AY757405,** *KF977435* | *KF977470* | **AY757481,** *KF977447* |
| *Carex deweyana* | **AY757412** | **HQ589994** | **AY757475** |
| *Carex intumescens* | **AY757579,** *KF977436* | *KF977471* | **AY757506,** *KF977448* |
| *Carex pensylvanica* | **AY757622** | **HQ590006, HQ590007** | **AY757550** |
| *Carex projecta* | **AY757423,** *KF977437* | *KF977472* | **AY757487,** *KF977449* |
| *Cinna latifolia* | **FJ766145,** *KF977438* | *KF977473* | **GQ324396,** *KF977450* |
| *Dryopteris intermedia* | *KF977439* | **AF537229,** *KF977474* | **FR731993, FR731994,** *KF977451* |
| *Gymnocarpium dryopteris* | NA | **HQ676503,** *KF977475* | **HQ676528,** *KF977452* |
| *Linaria vulgaris* | **DQ531053** | **HQ590160** | **AY492181** |
| *Linnaea borealis* | **AY236181** | **AF446941** | **AF366912** |
| *Lycopodium annotinum* | **AF338755,** *KF977441* | **AB574622,** *KF977477* | *KF977453* |
| *Lycopodium clavatum* | **AF338759** | **DQ026595,** *KF977478* | *KF977454* |
| *Lycopodium obscurum* | **AF338752** | **AB574634,** *KF977479* | *KF977455* |
| *Maianthemum canadense* | **EU850029** | **HQ590172** | **EU850199** |
| *Mitchella repens* | **AF072019** | **AF190440** | **FJ906973** |
| *Oryzopsis asperifolia* | **GU254653** | **HQ590194** | **GU254964** |
| *Ostrya virginiana* | **AF432064** | **X56620** | **AY211425** |
| *Phegopteris connectilis* | NA | **JF832080,** *KF977480* | **HQ676531,** *KF977456* |
| *Pinus strobus* | **AF036982** | **AY497219** | **DQ010640** |
| *Poa pratensis* | **FJ766176** | **HQ590213** | **AY504641** |
| *Polygala paucifolia* | **GQ888996** | **HQ590215** | **GQ888849** |
| *Polygonatum pubescens* | NA | **HQ590216,** *KF977462* | *KF977457* |
| *Pteridium aquilinum* | **X91872** | **AY300097** | **AY300044** |
| *Quercus rubra* | **AF098418** | **M58391** | **FJ490789** |
| *Rubus*  *allegheniensis* | **AF055772, AF055773,** *KF977442* | **EU676983, EU676984, EU676985** | *KF977458* |
| *Schizachne purpurascens* | **FM179432,** *KF977434* | **HQ590262,** *KF977469* | *KF977446* |
| *Streptopus lanceolatus* | NA | **D17381,** *KF977463* | *KF977459* |
| *Trientalis borealis* | **AY855156** | **HQ590306** | **AY855131** |
| *Tsuga canadensis* | NA | **AY056581,** *KF977464* | **EF395420,** *KF977460* |
| *Veronica officinalis* | **AF313012** | **AY034024** | **AF486391** |
| *Viola laboradorica* | *KF977431* | *KF977465* | *KF977461* |
